# Change of sexual behavior among men who have sex with men before, during and after COVID-19 pandemic in China: a cross-sectional study

**DOI:** 10.1186/s12879-023-08488-2

**Published:** 2023-08-10

**Authors:** Shi Wang, Jie Yang, Dandan Niu, Yushan Hou, Liping Fei, Hehe Zhao, Fangfang Chen, Fan Lv

**Affiliations:** 1 National Center for AIDS/STD Control and Prevention, Chinese Center for Disease Control and Prevention, Beijing, 102206 China; 2 Shenlan Public Health Counsel Service Center, Tianjin, China

**Keywords:** HIV, COVID-19, Sexual behavior, Men who have sex with men, Local epidemic, China

## Abstract

**Introduction:**

The COVID-19 epidemic control and prevention strategies affected people’s sexual activities and behaviors. Little was known about long-term effects of COVID-19 prevention and control strategies on sexual behaviors among men who have sex with men (MSM). This study aimed to examine changes in risky sexual behaviors of MSM before and after the local epidemic.

**Methods:**

An online survey was conducted nationwide from June 1 to June 10, 2022. MSM aged 16 years and above, residing in China were recruited through convenience sampling. A generalized estimating equation model with modified Poisson regression was used to analyze changes in multiple sexual partners, unprotected sex, mobility for sexual activity, and recreational substance use before and after the local epidemic.

**Results:**

Compared to the pre-pandemic (36.5%), the prevalence of multiple sexual partners (11.5%) significantly decreased during the local epidemic and then increased after the local epidemic (25.2%) but remained lower than pre-pandemic, as did the prevalence of unprotected sex (31.1%, 19.4%, and 26.1%), mobility for sexual activity (7.5%, 2.8%, and 4.1%) and recreational substance use (47.7%, 27.2%, and 39.5%). Compared to the pre-pandemic, higher declines in the prevalence of risky sexual behaviors during the local epidemic existed among MSM living without a regular partner (44% decrease in unprotected sex and 46% in recreational substance use), with a bachelor’s degree and above (70% decrease in multiple sex partners, 39% in unprotected sex, 67% in mobility for sexual activity and 44% in recreational substance use), higher incomes (70% decrease in multiple sex partners), self-identified gay or bisexual/unsure (38-71%), and HIV infection (49-83% decrease respectively in these four indicators). After the local epidemic, the declines in the above indicators compared to the pre-pandemic were correspondingly. And higher declines existed among MSM living without a regular partner (8% decrease in unprotected sex and 13% in recreational substance use), with a bachelor’s degree and above (33% decrease in multiple sex partners), higher incomes (55% decrease in mobility for sexual activity), self-identified gay (51% decrease in mobility for sexual activity), and HIV infection (32%, 68%, 24% decrease respectively in unprotected sex, mobility for sexual activity and recreational substance use).

**Conclusions:**

Risky sexual behaviors reduced considerably during the local epidemic, then seemed rebounded after the outbreak but wouldn’t return to pre-pandemic levels. More attention should be paid to vulnerable people with lower socio-economic status, HIV-positive, and sexual minorities for sustained HIV and COVID-19 prevention.

**Supplementary Information:**

The online version contains supplementary material available at 10.1186/s12879-023-08488-2.

## Introduction

The World Health Organization (WHO) estimated that as of October 18, 2022, COVID-19 has affected more than 200 countries and territories, with more than 620 million confirmed cases and more than 6.5 million deaths [1]. The outbreak mitigation strategies including physical isolation or stay-at-home orders effectively stopped the spread of COVID-19, but somewhat suppressed people’s sexual desires, resulting in a decline in sexual activity and behavior [2, 3]. Additionally, COVID-19 could also be regarded as a traumatic stressor event capable of affecting people’s sexual function and activity through a strong bidirectional relationship between mental health and sexual health [4, 5].

Men who have sex with other men (MSM) have long been considered more vulnerable to HIV infection due to the ongoing risk behaviors, such as unprotected receptive anal intercourse, high frequency of male partners, high number of lifetime male partners, etc. [6]. MSM may continue to engage in risky sexual behaviors regardless of stay-at-home orders and face barriers to access HIV and sexual health services during the COVID-19 epidemic [7]. These might put them at dual risk for HIV and COVID-19 transmission or acquisition. Although some studies reported fewer sexual partners and decreases in sexual behaviors among MSM during the outbreak of COVID-19, compared to the pre-pandemic [8–13], little is known about the long-term changes in their sexual behaviors over time during the COVID-19 epidemic [14, 15].

In late January 2020, China adopted strict suppression and containment strategies to stop indigenous transmission of COVID-19 [16]. By 7 May 2020, most cities returned to normal work and life [17]. The “Dynamic COVID-zero” policy was then adopted to balance the prevention and control of the epidemic with socioeconomic stability [18]. In this study, we conducted a national online survey to examine the changes in sexual risk behaviors of MSM over time before and after the COVID-19 pandemic, which would have implications for optimizing HIV-related service delivery and enhancing HIV prevention efforts during the epidemic.

## Methods

### Study design and participants

A cross-sectional survey was performed between 1 June and 10 June 2022, to assess the impact of COVID-19 on sexual health among MSM in the country. Participants were recruited by trained MSM-led community-based organizations (CBOs) staff through convenience sampling. Recruitment strategies included sharing QR codes or links to questionnaires on homosexual social media apps (e.g., Blued, etc.) and public social media platforms (WeChat and QQ), as well as recruiting offline at bars or parks, etc. We aimed to enroll 7000 eligible MSM covering 30 provinces (except Tibet, where no CBO has been successfully contacted before the survey) and the sample size was proportional to the estimated MSM population size for each province [19]. The numbers of participants enrolled in each region were 1174 in North China, 590 in Northeast China, 2225 in East China, 915 in Central China, 618 in South China, 807 in Southwest China, and 698 in Northwest China. All participants read the informed consent form and clicked ‘yes’ to begin the survey on the online questionnaire platform Wenjuanxing (www.wjx.com). Inclusion criteria were men aged 16 years or older, having sex with men before COVID-19, residing in China, and those willing and able to take this online survey. A pilot survey of 30 MSM was carried out to test the questionnaire before the formal study.

### Measures

We collected HIV status and sociodemographic information including age, residential status, education, average monthly personal income, living place, and sexual orientation. For residential status, we were concerned with whether the participants lived with a regular sex partner or spouse. Participants were questioned about risky sexual behaviors at three-time points, including pre-pandemic, during, and after the local epidemic. Four risk behaviors that were predictors of HIV transmission included the number of sexual partners, condom use, mobility for sexual activity, and recreational substance use.

We defined pre-pandemic period to be the time before January 2020 [20]. The period during the local epidemic was defined as the outbreak of the last local epidemic (having at least one confirmed case of SARS-CoV-2 infection in the county) with restrictions on public gatherings, entering and exiting the residential areas or neighborhood since May 2020 [17]. The period after the local epidemic was defined as the end of the last local epidemic with containment measures removed.

Participants were asked about the average number of sexual partners per month (0, 1, 2–3, 4–5, 6 and more) at three-time points. For those having more than two sexual partners on the monthly average, we defined as sex with multiple partners. Referring to the frequency of condom use during sexual intercourse (never, sometimes, or always), we defined sexual intercourse without or inconsistent condom use as unprotected sex. Also, we asked about mobility for sexual activity (having sex within or outside the current city of residence) and recreational substance use during sexual intercourse.

### Statistical analyses

Descriptive statistics were used to summarize the demographics and HIV status of the study population and compare changes in risky sexual behaviors at three-time points. Generalized estimating equations with modified Poisson regression (i.e., with robust standard errors) were performed to estimate the likelihood of reporting outcomes during and after the local pandemic compared with pre-pandemic. The overestimation of prevalence ratios for nonrare binary outcomes can be corrected using this method, which also helps to account for repeated measurements within individuals [21]. All models were adjusted for self-reported outcome indicator variables from the pre-pandemic. Regression models stratified by demographics were also generated. The Wald test was performed for interaction terms between time and these demographic factors to assess differences among subgroups of the same variable. All statistical tests were two-sided with p < 0.05 as the level of significance. Statistical analyses were done with R software (version 4.0.2).

## Results

### Study Population

From June 1 to June 10, 2022, 13,300 clicked the questionnaire and 7646 finished the questionnaire. A total of 91.9% (7027/7646) respondents was included in the analysis. The median age was 30 years (interquartile range: 25–36 years) and 48.4% (3404/7027) were aged 16–29 years, 75.7% (5320/7027) were living without a regular partner, 76.6% (5386/7027) had a bachelor’s degree or higher education, 6.2% (438/7027) resided in rural areas; 76.3% (5360/7027) self-identified as gay and 11.7% (824/7027) were HIV-positive (Table 1). The proportions of MSM without sexual intercourse were 40.2% and 14.0% during and after the local epidemic and varied across subgroups (Table S1-S2).

**Table 1:** Sociodemographic characteristics of an online sample of men who have sex with men (MSM) (n = 7027)

Characteristics	n	Percentage (%)/Interquartile range
Median age (years)	30	25–36
Age group		
16–29	3404	48.4
30–39	2490	35.4
≥ 40	1133	16.1
Whether living with a regular sexual partner or spouse or not		
No	5320	75.7
Yes	1707	24.3
Education		
Secondary school or less	414	5.9
High school	1227	17.5
College or higher	5386	76.6
Average monthly personal income ^†^		
< 1000 RMB	766	10.9
1000–5000 RMB	2671	38.0
5000–10,000 RMB	2357	33.5
≥ 10,000 RMB	1233	17.5
Living place		
Urban	6589	93.8
Rural	438	6.2
Sexual orientation		
Heterosexuality	277	3.9
Gay/homosexual	5360	76.3
Bisexual/unsure	1390	19.8
HIV status		
HIV negative/unknown	6203	88.3
HIV positive	824	11.7

Note: † Monthly income measured in Chinese Yuan

### Change in multiple sex partners

The prevalence of multiple sex partners was 36.5% before the epidemic, then fell to 11.5% and 25.2% during and after the epidemic (Table 2). The regression model showed that the prevalence of multiple partners was significantly lower during the local epidemic compared to pre-pandemic [adjusted prevalence ratio (aPR): 0.315, 95% confidence interval (CI): 0.297–0.335]. Although the prevalence increased after the local epidemic, it was still significantly lower than the pre-pandemic (aPR: 0.691, 95% CI: 0.668–0.714). Changes in the prevalence of multiple sexual partners were similar across all groups stratified by demographics and HIV status.

**Table 2:** Change in multiple sex partners before, during and after the pandemic

	Prevalence (%)				aPR (95%CI) ^†^	
	Pre-pandemic	During the local epidemic	After the local epidemic		During the local epidemic vs. Pre-pandemic	After the local epidemic vs. Pre-pandemic
Overall	36.5	11.5	25.2		0.315 (0.297–0.335)	0.691 (0.668–0.714)
Age group						
16–29	36.1	12.3	24.9		0.340 (0.313–0.370)	0.690 (0.657–0.725)
30–39	36.9	11.8	26.0		0.321 (0.291–0.355)	0.705 (0.668–0.744)
≥ 40	36.6	8.4	24.2		0.229 (0.191–0.275) ***	0.660 (0.607–0.718)
Whether living with a regular sexual partner or spouse or not						
No	37.9	11.7	26.2		0.308 (0.287–0.330)	0.690 (0.665–0.717)
Yes	32.0	11.0	22.1		0.342 (0.303–0.387)	0.692 (0.645–0.743)
Education						
Secondary school or less	40.6	16.4	32.1		0.405 (0.329–0.497)	0.792 (0.700-0.896)
High school	39.2	13.0	28.4		0.331 (0.298–0.378)	0.726 (0.674–0.781)
College or higher	35.5	10.8	23.9		0.304 (0.282–0.326) **	0.673 (0.647-0.700) *
Average monthly personal income						
< 1000 RMB	33.0	12.5	23.2		0.379 (0.319–0.451)	0.704 (0.631–0.784)
1000–5000 RMB	38.3	11.4	26.3		0.297 (0.269–0.329) *	0.688 (0.653–0.725)
5000–10,000 RMB	35.7	10.7	24.2		0.301 (0.270–0.335) *	0.679 (0.640–0.720)
≥ 10,000 RMB	36.3	12.6	25.8		0.347 (0.303–0.397)	0.711 (0.659–0.768)
Living place						
Urban	36.3	11.2	24.9		0.307 (0.288–0.327)	0.686 (0.663–0.710)
Rural	38.4	16.4	29.0		0.429 (0.351–0.523) **	0.756 (0.668–0.856)
Sexual orientation						
Heterosexuality	30.3	17.0	22.4		0.560 (0.454–0.690)	0.738 (0.614–0.887)
Gay/homosexual	37.2	10.9	25.5		0.292 (0.272–0.314) ***	0.687 (0.661–0.713)
Bisexual/unsure	35.0	12.9	24.5		0.368 (0.324–0.417) ***	0.698 (0.647–0.754)
HIV status						
HIV negative/unknown	36.4	11.8	25.4		0.325 (0.306–0.347)	0.698 (0.674–0.723)
HIV positive	37.4	9.0	23.7		0.240 (0.196–0.295) **	0.633 (0.572–0.701)

Note: Prevalence ratios calculated using generalized estimating equations and Poisson regression with robust standard errors to account for repeated measures among individuals and adjusting for reported value during the pre-pandemic

*p < 0.05; ** p < 0.01; *** p < 0.001

† The p _*interaction*_ values were calculated from Wald tests for interaction terms between time and these demographic factors

Abbreviations: aPR, adjusted prevalence ratio; CI, confidence interval; MSM, men who have sex with men

Compared to pre-pandemic, MSM aged 40 years and older (aPR: 0.229, 95% CI: 0.191–0.275), having a bachelor’s degree and above (aPR: 0.304, 95% CI: 0.282–0.326) or living in urban areas (aPR: 0.307, 95% CI: 0.288–0.327) had greater reductions in the prevalence of multiple partners during the local epidemic. The greater reductions also existed among MSM with an average monthly income of 1000–5000 RMB (aPR. 0.297, 95% CI: 0.269–0.329) or 5,000–10,000 RMB (aPR: 0.301, 95% CI: 0.270–0.335), being self-identified as gay (aPR: 0.292, 95% CI: 0.272–0.314) or bisexual/unsure (aPR: 0.368, 95% CI: 0.324–0.417), or being HIV-positive (aPR: 0.240, 95% CI: 0.196–0.295). Those with a bachelor’s degree or higher education (aPR: 0.673, 95% CI: 0.647-0.700) reported a greater reduction in the prevalence of multiple partners after the local epidemic than the pre-pandemic level.

### Change in unprotected sex

The prevalence of unprotected sex decreased during and after the local epidemic compared to pre-pandemic (31.1%), at 19.4% and 26.1% respectively (Table 3). According to the regression model, the prevalence of unprotected sex was statistically lower during the local epidemic than it was before (aPR: 0.624, 95% CI: 0.601–0.649). The prevalence was higher after the local epidemic but was still below the pre-pandemic level (aPR: 0.840, 95% CI: 0.817–0.865). Changes in the prevalence of unprotected sex were similar across all subgroups.

**Table 3:** Change in unprotected sex before, during and after the pandemic

	Prevalence (%)				aPR (95%CI) ^¶^	
	Pre-pandemic ^†^	During the local epidemic ^‡^	After the local epidemic ^§^		During the local epidemic vs. Pre-pandemic	After the local epidemic vs. Pre-pandemic
Overall	31.1	19.4	26.1		0.624 (0.601–0.649)	0.840 (0.817–0.865)
Age group						
16–29	31.2	19.0	26.0		0.613 (0.579–0.649)	0.833 (0.797–0.871)
30–39	30.4	20.0	26.2		0.660 (0.620–0.703)	0.863 (0.825–0.903)
≥ 40	32.5	19.0	26.4		0.583 (0.529–0.643)	0.815 (0.763–0.870)
Whether living with a regular sexual partner or spouse or not						
No	29.1	16.2	23.3		0.561 (0.533–0.591)	0.804 (0.775–0.834)
Yes	37.5	29.2	34.9		0.776 (0.735–0.819) ***	0.928 (0.889–0.969) ***
Education						
Secondary school or less	37.6	27.2	32.3		0.715 (0.633–0.808)	0.853 (0.779–0.933)
High school	34.8	22.9	28.9		0.662 (0.610–0.718)	0.834 (0.783–0.888)
College or higher	29.8	18.0	25.0		0.606 (0.578–0.635) *	0.841 (0.813–0.870)
Average monthly personal income						
< 1000 RMB	33.2	19.8	26.8		0.604 (0.536–0.682)	0.821 (0.750–0.899)
1000–5000 RMB	30.8	18.7	25.7		0.610 (0.571–0.651)	0.835 (0.796–0.876)
5000–10,000 RMB	31.0	20.6	27.2		0.668 (0.628–0.712)	0.879 (0.838–0.922)
≥ 10,000 RMB	30.9	18.0	24.5		0.584 (0.531–0.642)	0.790 (0.738–0.846)
Living place						
Urban	30.6	19.1	25.8		0.626 (0.602–0.652)	0.845 (0.821–0.871)
Rural	39.8	23.8	31.0		0.600 (0.520–0.692)	0.781 (0.705–0.866)
Sexual orientation						
Heterosexuality	30.7	24.4	26.9		0.788 (0.666–0.933)	0.883 (0.769–1.015)
Gay/homosexual	31.5	19.5	26.2		0.620 (0.593–0.648) **	0.829 (0.802–0.857)
Bisexual/unsure	29.6	18.0	25.7		0.608 (0.556–0.665) **	0.878 (0.826–0.932)
HIV status						
HIV negative/unknown	30.4	19.7	26.3		0.649 (0.624–0.676)	0.866 (0.841–0.893)
HIV positive	36.7	16.9	24.8		0.467 (0.410–0.531) ***	0.677 (0.619–0.740) ***

Note: Prevalence ratios calculated using generalized estimating equations and Poisson regression with robust standard errors to account for repeated measures among individuals and adjusting for reported value during the pre-pandemic

*p < 0.05; ** p < 0.01; *** p < 0.001

† 153 MSM missing values of unprotected sex were excluded in the analysis at pre-pandemic period

‡ 187 MSM missing values of unprotected sex were excluded in the analysis during the local epidemic

§ 166 MSM missing values of unprotected sex were excluded in the analysis after the local epidemic

¶ The p _*interaction*_ values were calculated from Wald tests for interaction terms between time and these demographic factors

Abbreviations: aPR, adjusted prevalence ratio; CI, confidence interval; MSM, men who have sex with men

Compared to the pre-pandemic, those living without a regular partner (aPR: 0.561, 95% CI: 0.533–0.591), undergraduate and above (aPR: 0.606, 95% CI: 0.578–0.635), self-identified as gay (aPR: 0.620, 95% CI: 0.593–0.648) or bisexual/unsure (aPR: 0.608, 95% CI: 0.556–0.665), and HIV-positive (aPR: 0.467, 95% CI: 0.410–0.531) showed greater reductions in the prevalence of unprotected sex during the local epidemic. However, the decreases after the local epidemic were higher among those living without a regular partner (aPR: 0.804, 95% CI: 0.775–0.834), and HIV-positive (aPR: 0.677, 95% CI: 0.619–0.740). The prevalence of unprotected sex declined after the local epidemic in subgroups of gay (aPR: 0. 829, 95% CI: 0.802–0.857) and bisexual/unsure (aPR: 0.878, 95% CI: 0.826–0.932) compared to pre-pandemic, but the difference was not statistically significant in heterosexual MSM.

### Change in mobility for sexual activity

During and after the local epidemic, fewer people migrated to other cities for sexual activity than pre-pandemic (7.5%), with rates of 2.8% and 4.1%, respectively (Table 4). The regression model revealed that the prevalence of mobility reduced during the local epidemic (aPR: 0.374, 95% CI: 0.330–0.424), and although the prevalence increased after the local epidemic, it was still significantly lower than the pre-pandemic (aPR: 0.537, 95% CI: 0.485–0.594). Changes in the prevalence of mobility were similar across all subgroups.

**Table 4:** Change in mobility for sexual activity before, during and after the pandemic

	Prevalence (%)				aPR (95%CI) ^¶^	
	Pre-pandemic ^†^	During the local epidemic ^‡^	After the local epidemic ^§^		During the local epidemic vs. Pre-pandemic	After the local epidemic vs. Pre-pandemic
Overall	7.5	2.8	4.1		0.374 (0.330–0.424)	0.537 (0.485–0.594)
Age group						
16–29	7.9	3.1	4.4		0.392 (0.330–0.464)	0.552 (0.480–0.634)
30–39	7.2	2.5	3.8		0.363 (0.290–0.453)	0.516 (0.431–0.617)
≥ 40	7.1	2.4	3.8		0.341 (0.243–0.478)	0.533 (0.409–0.695)
Whether living with a regular sexual partner or spouse or not						
No	8.0	3.0	4.5		0.375 (0.326–0.432)	0.561 (0.503–0.627)
Yes	6.1	2.2	2.7		0.372 (0.279–0.494)	0.437 (0.341–0.560)
Education						
Secondary school or less	8.6	5.2	5.6		0.625 (0.437–0.893)	0.674 (0.496–0.916)
High school	7.5	3.6	5.2		0.480 (0.376–0.613)	0.668 (0.553–0.807)
College or higher	7.5	2.4	3.7		0.328 (0.280–0.385) **	0.495 (0.436–0.561)
Average monthly personal income						
< 1000 RMB	7.6	3.4	5.0		0.451 (0.312–0.651)	0.651 (0.490–0.866)
1000–5000 RMB	7.5	3.0	4.1		0.403 (0.330–0.492)	0.552 (0.467–0.652)
5000–10,000 RMB	7.2	2.2	3.3		0.316 (0.251–0.399)	0.452 (0.375–0.545) *
≥ 10,000 RMB	8.4	3.1	4.9		0.372 (0.277–0.498)	0.585 (0.469–0.730)
Living place						
Urban	7.1	2.5	3.8		0.363 (0.316–0.417)	0.529 (0.474–0.590)
Rural	14.2	6.5	8.6		0.456 (0.337–0.616)	0.598 (0.466–0.766)
Sexual orientation						
Heterosexuality	18.1	10.1	12.7		0.559 (0.426–0.733)	0.693 (0.548–0.877)
Gay/homosexual	7.0	2.3	3.4		0.335 (0.285–0.395) **	0.490 (0.429–0.559) *
Bisexual/unsure	7.7	3.3	4.9		0.422 (0.327–0.547)	0.630 (0.520–0.762)
HIV status						
HIV negative/unknown	7.3	3.0	4.2		0.407 (0.357–0.463)	0.573 (0.516–0.636)
HIV positive	9.2	1.5	2.9		0.172 (0.102–0.288) **	0.315 (0.218–0.456) **

Note: Prevalence ratios calculated using generalized estimating equations and Poisson regression with robust standard errors to account for repeated measures among individuals and adjusting for reported value during the pre-pandemic

*p < 0.05; ** p < 0.01; *** p < 0.001

† 182 MSM missing values of mobility to other cities were excluded in the analysis at pre-pandemic period

‡ 249 MSM missing values of mobility to other cities were excluded in the analysis during the local epidemic

§ 201 MSM missing values of mobility to other cities were excluded in the analysis after the local epidemic

¶ The p _*interaction*_ values were calculated from Wald tests for interaction terms between time and these demographic factors

Abbreviations: aPR, adjusted prevalence ratio; CI, confidence interval; MSM, men who have sex with men

Individuals who were undergraduate and above (aPR: 0.328, 95% CI: 0.280–0.385), self-identified as gay (aPR: 0.335, 95% CI: 0.285–0.395), and HIV-positive (aPR: 0.172, 95% CI: 0.102–0.288) showed higher decreases in mobility for sexual activity during the local epidemic. Those with an average monthly income of RMB 5,000–10,000 (aPR: 0.452, 95% CI: 0.375–0.545), self-identified as gay (aPR: 0.490, 95% CI: 0.429–0.559), and HIV-positive individuals (aPR: 0.315, 95% CI: 0.218–0.456) had larger declines after the local epidemic compared to the pre-pandemic.

### Change in recreational substance use

When compared to pre-pandemic (47.7%), recreational substance use was less common during and after the local epidemic (27.2% and 39.5%) (Table 5). In comparison to pre-pandemic levels, the regression model demonstrated a significant decline in recreational substance use during (aPR: 0.576, 95% CI: 0.559–0.594) and after the local epidemic (aPR: 0.832, 95% CI: 0.816–0.847). All subgroups experienced the same changes.

**Table 5:** Change in recreational substance use before, during and after the pandemic

	Prevalence (%)				aPR (95%CI) ^¶^	
	Pre-pandemic ^†^	During the local epidemic ^‡^	After the local epidemic ^§^		During the local epidemic vs. Pre-pandemic	After the local epidemic vs. Pre-pandemic
Overall	47.7	27.2	39.5		0.576 (0.559–0.594)	0.832 (0.816–0.847)
Age group						
16–29	47.6	27.1	38.8		0.575 (0.549–0.601)	0.817 (0.794–0.841)
30–39	51.0	29.7	42.6		0.585 (0.557–0.614)	0.837 (0.814–0.861)
≥ 40	40.7	22.2	35.0		0.558 (0.513–0.606)	0.868 (0.829–0.908) *
Whether living with a regular sexual partner or spouse or not						
No	47.4	25.3	38.7		0.539 (0.519–0.560)	0.820 (0.802–0.839)
Yes	48.7	33.1	42.1		0.687 (0.654–0.722) ***	0.866 (0.838–0.895) **
Education						
Secondary school or less	43.1	28.5	37.7		0.675 (0.603–0.755)	0.883 (0.816–0.955)
High school	45.5	28.7	39.3		0.636 (0.595–0.681)	0.868 (0.830–0.907)
College or higher	48.6	26.8	39.7		0.557 (0.537–0.577) **	0.821 (0.803–0.838)
Average monthly personal income						
< 1000 RMB	43.5	22.5	34.5		0.520 (0.465–0.581)	0.797 (0.743–0.854)
1000–5000 RMB	44.4	25.2	36.4		0.575 (0.545–0.605)	0.825 (0.798–0.852)
5000–10,000 RMB	50.1	29.3	42.3		0.591 (0.562–0.622) *	0.845 (0.820–0.871)
≥ 10,000 RMB	52.9	30.4	44.1		0.580 (0.541–0.622)	0.838 (0.806–0.871)
Living place						
Urban	47.8	27.2	39.7		0.575 (0.557–0.594)	0.835 (0.819–0.851)
Rural	46.7	27.5	36.3		0.593 (0.525–0.670)	0.781 (0.714–0.855)
Sexual orientation						
Heterosexuality	38.8	26.9	32.6		0.725 (0.634–0.830)	0.841 (0.761–0.930)
Gay/homosexual	48.4	27.2	40.1		0.567 (0.548–0.588) ***	0.831 (0.813–0.849)
Bisexual/unsure	46.8	27.1	38.7		0.588 (0.549–0.630) **	0.834 (0.800–0.870)
HIV status						
HIV negative/unknown	46.2	26.8	38.8		0.587 (0.568–0.606)	0.843 (0.827–0.860)
HIV positive	59.5	30.1	45.2		0.512 (0.468–0.561) **	0.763 (0.721–0.807) **

Note: Prevalence ratios calculated using generalized estimating equations and Poisson regression with robust standard errors to account for repeated measures among individuals and adjusting for reported value during the pre-pandemic

*p < 0.05; ** p < 0.01; *** p < 0.001

† 181 MSM missing values of recreational substance use were excluded in the analysis of the pre-pandemic period

‡ 159 MSM missing values of recreational substance use were excluded in the analysis during the local epidemic

§ 172 MSM missing values of recreational substance use were excluded in the analysis after the local epidemic. The p _*interaction*_ values were calculated from Wald tests for interaction terms between time and these demographic factors

Abbreviations: aPR, adjusted prevalence ratio; CI, confidence interval; MSM, men who have sex with men

MSM living without a regular partner (aPR: 0.539, 95% CI: 0.519–0.560), having a bachelor’s degree or higher (aPR: 0.557, 95% CI: 0.537–0.577), earning an average monthly income of less than 1,000 RMB (aPR: 0.520, 95% CI: 0.465–0.581), self-identified as gay (aPR: 0.567, 95% CI: 0.548–0.588) or bisexual/unsure (aPR: 0.588, 95% CI: 0.549–0.630), and being HIV positive (aPR: 0.512, 95% CI: 0.468–0.561) showed significant reductions in recreational substance use during the local epidemic compared to pre-pandemic. After the local epidemic, people aged 16–29 years (aPR: 0.817, 95% CI: 0.794–0.841), living without a regular partner (aPR: 0.820, 95% CI: 0.802–0.839) and being HIV-positive (aPR: 0.763, 95% CI: 0.721–0.807) had a larger decrease in recreational substance use compared to the pre-pandemic.

## Discussion

This study was a nationwide survey on changes in sexual behavior among MSM at three time periods of COVID-19. Our findings confirmed a significant decline in the prevalence of multiple sexual partners, unprotected sex, mobility for sexual activity, and recreational substance use among MSM in China during the local epidemic, which was consistent with prior studies [8–13]. This might be related to the strict COVID-19 mitigation measures such as social distancing implemented when the outbreak occurred locally [2, 16]. Nevertheless, we also found that the prevalence of sexual activities resumed after the local epidemic control and with quarantine measures lifted, in line with the results of a US cohort study [14], but would not quickly get back to the pre-pandemic level. Few studies have discussed this. In our opinion, elevated rates of distress, anxiety, and PTSD-like symptoms related to the pandemic might negatively affect sexual practices [4]. It is uncertain whether the gap in sexual activities would be persistent after the pandemic in the long term. However, the adverse impact of this potential “rebound” in sexual risk warranted attention, particularly for areas hit hard by the epidemic and at risk of persistent disruption of clinical services. These changes were also observed across subgroups by age, residence status, education level, income, urban/rural, sexual orientation, and HIV status.

Containment measures to maintain social distance would directly affect the residence status of MSM. It was observed that MSM living without a regular partner experienced lower rates of unprotected sex and recreational substance use during and after the local epidemic, which might be caused by the high levels of no sex at the two periods in the study. Previous studies also found a decline in the frequency of sexual activity or even temporary abstinence in MSM during the pandemic, with a greater reduction in sex with casual partners than with regular partners [9, 14]. The high proportion of no sex among MSM living without a regular partner might be caused by their personal health issues and a sense of communal altruism which referred to reduced sexual behavior for the goal of for the purpose of protecting vulnerable people they lived with (e.g., immunocompromised parents or sexual partners) from COVID-19 and HIV infections [22, 23].

Notably, the COVID-19 pandemic exacerbated structural inequalities particularly for MSM with lower socioeconomic status (SES) which increased their risk of HIV and COVID-19 infection [24]. Our study showed that people with higher education and wage levels experienced a greater decline during the pandemic in multiple sex partners, unprotected sex, and mobility for sexual activity. Because those with high SES were prone to have higher risk awareness and increase self-protective behaviors [2]. In contrast, individuals of a lower SES may suffer disproportionately from higher unemployment rates during the COVID-19 crisis, which increased the risk of poorer mental health and propensity for risky sexual behaviors [8, 25]. Moreover, a greater reduction in recreational substance use existed in low-income people because of the further fall in their disposable income during the pandemic [26].

Reassuringly, we found that during and after the local epidemic, sexual minorities and HIV infections had greater reductions in the four indicators of risky sex, potentially implying a reduced risk of infection and transmission of HIV and COVID-19. This might be associated with their fear of being stigmatized or discriminated against, as well as contracting COVID-19 due to their HIV status [27]. MSM who required their partners to engage in preventive activities like cruising outside and glory holes during the pandemic could limit face-to-face contact and lower their chance of COVID-19 infection. People living with HIV were more likely to adopt strict strategies to reduce risk, such as avoiding crowded places, casual sex, and group sex events [9]. But we must equally be aware that it still has unsettling ramifications. Before the COVID-19 epidemic, MSM had experienced rejection and additional pressure due to lack of understanding from their families [28]. Physical distancing measures may increase social isolation for them compared to pre-pandemic [29]. Due to social discrimination and the protection of individual privacy in epidemiological investigation, homosexual and bisexual people were often going out for necessities instead of having sexual behaviors during the pandemic, which suppressed the sexual desires of MSM. Because of lacking social support and excessive repression, MSM may be still at risk of rebounding to high-risk sexual behaviors in the later stages of the pandemic[14].

In our analysis, MSM with older age and living in urban areas had a greater reduction in multiple sex partners during the pandemic. Older people were more likely to seek sexual partners offline dating (baths, bars, parks, etc.) instead of online dating applications. During the epidemic, offline dating has also been severely affected, causing a greater decline in the prevalence of multiple partners [7]. Compared with rural-dwelling MSM, those dwelling in urban might face high burden of COVID-19 epidemic and also more easily get access to information about COVID-19 and have better compliance with social distancing and stay-at-home measures, which was supposed to greatly reduce their multiple partners during the epidemic [30].

This is the first nationwide study to report the persistent changes in sexual behavior among MSM in China in the COVID-19 era. The findings would be helpful to better understand the variation of sexual characteristics among MSM during the epidemic by comparing changes in sexual partners, mobility for sexual activity, condom use, and recreational substance use before, during, and after the epidemic. Further study combined with our behavioral and testing data is underway to assess the impact on HIV infection among MSM during the implementation of physical isolation and relaxation of restrictions. However, our study still has several limitations. First, the study may suffer from over-sampling owing to 90% of participants residing in urban cities especially capital cities, which may magnify the proportion of HIV-positive MSM (11.7%), much greater than the HIV prevalence of this population in the country (5.7%) [31]. The magnitude of change in their sexual behaviors should be interpreted cautiously, which may mostly represent the characteristics of MSM in urban areas. Second, the information on sexual behaviors at three-time points was collected simultaneously, which may be affected by recall bias and social desirability bias. Longitudinal studies are further needed to validate the pattern of sexual behaviors at three periods in our study. Finally, the measures relied on self-reporting, thus unavoidably leading to reporting bias. However, the information was collected through a web-based questionnaire and all participants were recruited anonymously by trained CBO staff, which made the participants more willing to participate and tell their true situation.

## Conclusions

Our study provided important information on trends in sexual behaviors of multiple partners, unprotected sex, mobility for sexual activity, and recreational substance use among MSM before and after the pandemic. Risky sexual behaviors have declined substantially compared to the pre-pandemic and rebounded after the local epidemic but did not quickly return to pre-pandemic levels. Changes in sexual behaviors varied by demographic characteristics and HIV status. Under the dual threat of HIV and COVID-19 infection and transmission, it is important to focus on vulnerable groups with lower SES, HIV-positive and sexual minorities, and provide targeted prevention measures.

### Electronic supplementary material

Below is the link to the electronic supplementary material.

Supplementary Material 1

## Data Availability

The datasets generated and analysed during the current study are not publicly available due to privacy or ethical restrictions but are available from the corresponding author on reasonable request.

## Declarations

### Authors’ information

National Center for AIDS/STD Control and Prevention, Chinese Center for Disease Control and Prevention, Beijing, China.

Shi Wang, Dandan Niu, Yushan Hou, Liping Fei, Hehe Zhao, Fangfang Chen, Fan Lv.

Shenlan Public Health Counsel Service Center, Tianjin, China.

Jie Yang.
