# Supplementary material for: Change of sexual behavior among men who have sex with men before, during and after COVID-19 pandemic in China: a cross-sectional study
Source: BMC Infect Dis. 2023 Aug 10;23:527. doi: 10.1186/s12879-023-08488-2 (PMC10416525; doi:10.1186/s12879-023-08488-2)
Supplement: Supplementary file 1 — Supplementary Material 1 [file 12879_2023_8488_MOESM1_ESM.docx]

**Supporting information**

**Table S1** Sexlessness in MSM populations during the local epidemic

|  | Have a sex | No sex | *P* value |
| --- | --- | --- | --- |
| Total | 4205 (59.8) | 2822 (40.2) |  |
| Age group |  |  | **0.012** |
| 16-29 | 2004 (58.9) | 1400 (41.1) |  |
| 30-39 | 1547 (62.1) | 943 (37.9) |  |
| ≥40 | 654 (57.7) | 479 (42.3) |  |
| Whether living with a regular sexual partner or spouse or not |  |  | **<0.001** |
| No | 2906 (54.6) | 2414 (45.4) |  |
| Yes | 1299 (76.1) | 408 (23.9) |  |
| Education |  |  | **<0.001** |
| Secondary school or less | 288 (69.6) | 126 (30.4) |  |
| High school | 773 (63.0) | 454 (37.0) |  |
| College or higher | 3144 (58.4) | 2242 (41.6) |  |
| Average monthly personal income |  |  | **0.005** |
| <1000 RMB | 422 (55.1) | 344 (44.9) |  |
| 1000-5000 RMB | 1577 (59.0) | 1094 (41.0) |  |
| 5000-10000 RMB | 1462 (62.0) | 895 (38.0) |  |
| ≥10000 RMB | 744 (60.3) | 489 (39.7) |  |
| Living place |  |  | 0.520 |
| Urban | 3936 (59.7) | 2653 (40.3) |  |
| Rural | 269 (61.4) | 169 (38.6) |  |
| Sexual orientation |  |  | **<0.001** |
| Heterosexuality | 205 (74.0) | 72 (26.0) |  |
| Gay/homosexual | 3147 (58.7) | 2213 (41.3) |  |
| Bisexual/unsure | 853 (61.4) | 537 (38.6) |  |
| HIV status |  |  | **0.001** |
| HIV negative/unknown | 3755 (60.5) | 2448 (39.5) |  |
| HIV positive | 450 (54.6) | 374 (45.4) |  |

**Table S2** Sexlessness in MSM populations after the local epidemic

|  | Have a sex | No sex | *P* value |
| --- | --- | --- | --- |
| Total | 6040 (86.0) | 987 (14.0) |  |
| Age group |  |  | **<0.001** |
| 16-29 | 2872 (84.4) | 532 (15.6) |  |
| 30-39 | 2195 (88.2) | 295 (11.8) |  |
| ≥40 | 973 (85.9) | 160 (14.1) |  |
| Whether living with a regular sexual partner or spouse or not |  |  | **<0.001** |
| No | 4449 (83.6) | 871 (16.4) |  |
| Yes | 1591 (93.2) | 116 (6.8) |  |
| Education |  |  | **0.008** |
| Secondary school or less | 374 (90.3) | 40 (9.7) |  |
| High school | 1069 (87.1) | 158 (12.9) |  |
| College or higher | 4597 (85.4) | 789 (14.6) |  |
| Average monthly personal income |  |  | **<0.001** |
| <1000 RMB | 616 (80.4) | 150 (19.6) |  |
| 1000-5000 RMB | 2316 (86.7) | 355 (13.3) |  |
| 5000-10000 RMB | 2053 (87.1) | 304 (12.9) |  |
| ≥10000 RMB | 1055 (85.6) | 178 (14.4) |  |
| Living place |  |  | **0.002** |
| Urban | 5686 (86.3) | 903 (13.7) |  |
| Rural | 354 (80.8) | 84 (19.2) |  |
| Sexual orientation |  |  | 0.114 |
| Heterosexuality | 246 (88.8) | 31 (11.2) |  |
| Gay/homosexual | 4583 (85.5) | 777 (14.5) |  |
| Bisexual/unsure | 1211 (87.1) | 179 (12.9) |  |
| HIV status |  |  | **<0.001** |
| HIV negative/unknown | 5374 (86.6) | 829 (13.4) |  |
| HIV positive | 666 (80.8) | 158 (19.2) |  |
